# Application of intraoperative lung-protective ventilation varies in accordance with the knowledge of anaesthesiologists: a single-Centre questionnaire study and a retrospective observational study

**DOI:** 10.1186/s12871-018-0495-7

**Published:** 2018-04-02

**Authors:** Seung Hyun Kim, Sungwon Na, Woo Kyung Lee, Hyunwoo Choi, Jeongmin Kim

**Affiliations:** Department of anesthesiology and Pain Medicine, anaesthesia and Pain Research Institute, Yonsei University College of Medicine, 50-1 Yonsei-ro, Seodaemun-gu, Seoul, 03722 South Korea

**Keywords:** Knowledge, Lung-protective ventilation, Mechanical ventilation, Perioperative ventilation

## Abstract

**Background:**

The benefits of lung-protective ventilation (LPV) with a low tidal volume (6 mL/kg of ideal body weight [IBW]), limited plateau pressure (< 28–30 cm H_2_O), and appropriate positive end-expiratory pressure (PEEP) in patients with acute respiratory distress syndrome have become apparent and it is now widely adopted in intensive care units. Recently evidence for LPV in general anaesthesia has been accumulated, but it is not yet generally applied by anaesthesiologists in the operating room.

**Methods:**

This study investigated the perception about intraoperative LPV among 82 anaesthesiologists through a questionnaire survey and identified the differences in ventilator settings according to recognition of lung-protective ventilation. Furthermore, we investigated the changes in the trend for using this form of ventilation during general anaesthesia in the past 10 years.

**Results:**

Anaesthesiologists who had received training in LPV were more knowledgeable about this approach. Anaesthesiologists with knowledge of the concept behind LPV strategies applied a lower tidal volume (median (IQR [range]), 8.2 (8.0–9.2 [7.1–10.3]) vs. 9.2 (9.1–10.1 [7.6–10.1]) mL/kg; *p* = 0.033) and used PEEP more frequently (69/72 [95.8%] vs. 5/8 [62.5%]; *p* = 0.012; odds ratio, 13.8 [2.19–86.9]) for laparoscopic surgery than did those without such knowledge. Anaesthesiologists who were able to answer a question related to LPV correctly (respondents who chose ‘height’ to a multiple choice question asking what variables should be considered most important in the initial setting of tidal volume) applied a lower tidal volume in cases of laparoscopic surgery and obese patients. There was an increase in the number of patients receiving LPV (V_T_ < 10 mL/kgIBW and PEEP ≥5 cm H_2_O) between 2004 and 2014 (0/818 [0.0%] vs. 280/818 [34.2%]; *p* <  0.001).

**Conclusions:**

Our study suggests that the knowledge of LPV is directly related to its implementation, and can explain the increase in LPV use in general anaesthesia. Further studies should assess the impact of using intraoperative LPV on clinical outcomes and should determine the efficacy of education on intraoperative LPV implementation.

**Electronic supplementary material:**

The online version of this article (10.1186/s12871-018-0495-7) contains supplementary material, which is available to authorized users.

## Background

Traditionally, anaesthesiologists have applied ventilation with tidal volumes (V_T_) between 10 and 15 mL/kg of body weight, and without positive end-expiratory pressure (PEEP), to prevent atelectasis [1, 2]. However, the concept of lung-protective ventilation (LPV) has recently emerged, based on previous studies that demonstrated the significant benefit of low V_T_ with appropriate PEEP on mortality in patients with acute respiratory distress syndrome (ARDS) [3]. Although the level of PEEP that balances alveolar recruitment against over-distension should be selected and titrated for individual patients [4–7], currently, LPV with low V_T_ (6 mL/kg of ideal body weight [IBW]), limited plateau pressure (< 28–30 cm H_2_O), and appropriate PEEP is generally accepted for ventilation in patients with ARDS.

Several studies have suggested the benefits of LPV during surgery [8, 9]. During laparoscopic surgery, LPV is associated with a relatively low incidence of pulmonary complications and better oxygenation [10–12]. The benefits of LPV have also been demonstrated in obese patients [13, 14]. In addition, Xiong et al. have reported that intraoperative LPV reduces barotrauma and lung inflammation in patients undergoing spinal surgery in the prone position [15, 16]. Overall, these findings highlight the advantage of using intraoperative LPV. There is an increasing amount of literature on intraoperative LPV patterns and trends. Although the traditional method of ventilation is still used [17–19], implementation of intraoperative LPV has increased [20]. According to a recent study, education and feedback decreased the average intraoperative tidal volume and improved the rate of LPV use [21].

In this questionnaire-based survey of anaesthesiologists, we focused on the effect of cognizance of intraoperative LPV strategies on the practical implementation of LPV in cases requiring general anaesthesia. In addition, by means of a retrospective study in a single university hospital in South Korea, we identified the factors that had influenced the changes in ventilation strategy over the past decade.

## Methods

### 1. Questionnaire survey of degree of recognition of lung-protective ventilation strategies

After obtaining approval from the relevant institutional review board, anaesthesiologists in a university hospital were recruited via email to participate in this questionnaire study in 2016. The investigators individually contacted the anaesthesiologists and enrolled them after obtaining written informed consent. A total of 82 anaesthesiologists—including 16 first- and second-year residents, 21 third- and fourth-year residents, 23 fellows, 9 assistant professors, 3 associate professors, and 10 professors—participated in the survey, which was designed to assess their cognizance of LPV and mechanical ventilation practices, including V_T_ settings and the application/non-application of PEEP. Respondents’ average clinical career was 2.6 years (residents), 7.8 years (fellows and assistant professors), and 23.4 years (associate professors and professors), respectively.

The questionnaire consisted of 6 questions (Additional file 1). The first 3 questions were about the ventilator settings (tidal volume and PEEP) that should be used in certain situations. Questions 1, 2, and 3 asked about ventilator settings in laparoscopic surgery, in non-laparoscopic surgery, and in obese patients, respectively. Question 4 asked if the respondents routinely applied PEEP in the initial ventilation setting. Respondents who answered ‘yes’ to Question 4 were considered to be applying PEEP routinely when setting up the ventilator.

Because knowing the approximate meaning is different from knowing the exact definition, we assumed that their answers about LPV may differ from their actual knowledge. Therefore, in the subsequent questions, we divided the respondents according to 2 criteria. Question 5 was a multiple-choice question asking what variables respondents consider most important in the initial setting of mechanical ventilation. From the LPV strategy perspective, the correct answer to this question is ‘height’ [22–24]; respondents who answered ‘height’ were classified as the ‘correct answer group’, and those who selected other answers were classified as the ‘incorrect answer group’. This classification was made irrespective of the response to question 6, which directly asked the respondents whether they knew about the LPV strategy. Respondents who replied ‘Yes’ to this question were considered as having knowledge of the concept of LPV, regardless of their answer to question 5, and were classified as the ‘conceptual group’, while respondents who answered ‘no’ were classified as the ‘non-conceptual group’.

First, we investigated whether the conceptual group and the non-conceptual group differed in terms of ventilator settings for laparoscopic surgery, non-laparoscopic surgery, and obese patients, and in the percentage of routine application of PEEP. Second, we determined whether the correct answer group and the incorrect answer group differed in terms of these aspects.

### 2. Retrospective study

#### Study population and data collection

After obtaining institutional review board approval and a waiver for obtaining informed patient consent, we queried our electronic medical records database for cases of surgery (in-patient surgery and day-of-surgery admission cases) under general anaesthesia at our university hospital between January 1, 2004 and December 31, 2004 and between January 1, 2014 and December 31, 2014. While 15,982 cases of surgery under general anaesthesia had been registered in 2004, the corresponding number of cases in 2014 exceeded 33,538. The exclusion criteria were as follows: age < 19 years; cardiac and thoracic surgery; insufficient medical data; more than 1 anaesthetic procedure during admission; preoperative ventilator care; and diagnosis of chronic obstructive lung disease or other respiratory diseases. For subgroup analysis, the patients were categorised by the type of surgery—laparoscopic, open abdominal, head and neck, orthopaedic, urological, spine, and other surgeries. Data regarding anaesthesia were retrieved from the electronic medical records.

#### Ventilator management and calculation of respiratory variables

Mechanical ventilation was applied using a variety of GE Healthcare (Madison, WI, USA) or Dräger (Drägerwerk AG, Lübeck, Germany) anaesthesia machines. During the study period, the following models were in use at our institution: Datex-Ohmeda and Avance Carestation (GE Healthcare); Jesus and Apollo (Dräger). The ventilator mode, PEEP, and ventilator settings (V_T_ and respiratory rate) were chosen at the discretion of the attending anaesthesiologist. For each patient, we recorded the initial values of expired V_T_, respiratory rate, and PEEP after induction of general anaesthesia. Only the initial settings were used for the analysis because there are many uncontrolled factors in such a retrospective study.

We calculated the V_T_/kg and V_T_/kgIBW; IBW was calculated using the following formula [18].

Male patients: IBW (kg) = 50 + 2.3 (height [inches] – 60).

Female patients: IBW (kg) = 45.5 + 2.3 (height [inches] – 60).

We defined a V_T_ > 10 mL/kgIBW and/or PEEP < 5 cmH_2_O as non-LPV [20, 25, 26], and PEEP ≥5 cmH_2_O as usage of PEEP [23], in accordance with the findings of previous studies.

#### Assessment of preoperative risk

General preoperative risk was assessed on the basis of ASA physical status score, age, sex, and body mass index (BMI).

#### Propensity-score matching

**Fig. 1:**
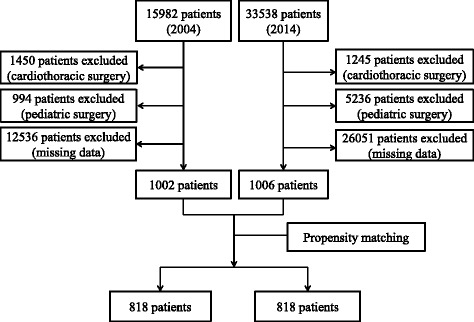
Recruitment flowchart of patients who underwent surgery under general anaesthesia in 2004 and 2014 at a single centre

Ventilator settings, such as V_T_ and PEEP, are determined on the basis of the height, weight, age, and sex of the patient [27]. Since our pre-analysis noted that there were significant differences in the height and sex between the 2004 and 2014 groups, a propensity score (PS)-matching technique was adopted to diminish the compounding effects of height and sex. The calculation of the PS involved the following: (1) using a logit model for matching the variables (height and sex) by considering the 2014 group as the treatment group and (2) predicting probabilities, termed PSs. PS-matching was implemented to pair the 2004 group with the 2014 group within a caliper of 0.01. There were no significant differences in height and sex between the 2004 and 2014 groups after completing PS-matching. In total, 818 matched patients in each group were used in the final analysis (Fig. 1).

#### Statistical analysis

Descriptive data are presented as mean values ± SD or median (IQR [range]). For intergroup comparisons, the chi-square test was used for categorical variables, and Student’s *t*-test or the Wilcoxon–Mann–Whitney test was used for continuous variables. Factors that affected V_T_ settings were determined by regression analysis. Multiple regression analysis included variables such as laparoscopic surgery, obesity, and prone position during surgery, which have been shown to affect LPV in previous studies [10–16]. All statistical analyses were performed using SPSS Statistics 23 (IBM SPSS Statistics for Windows, IBM Corp, Armonk, NY, USA).

## Results

### 1. Questionnaire survey

Application of LPV during general anaesthesia varied in accordance with the cognizance of LPV among the anaesthesiologists. While 73 respondents answered that they knew about LPV, 8 did not; 1 of the fellows had not responded to this question. In a multiple-choice question, 63 respondents chose the correct answer and 19 chose the wrong answer. There was no significant difference in the percentage of correctness between the conceptual group and non-conceptual group (58/73 [79.5%] vs. 5/8 [62.5%]; *p* = 0.367; odds ratio [OR] = 2.32 [95% CI, 0.50–10.82]).

#### Difference between the conceptual group and the non-conceptual group

Anaesthesiologists with knowledge of the concept of LPV (conceptual group) applied LPV more often during general anaesthesia than those without knowledge of LPV (non-conceptual group; Table 1). Among cases of laparoscopic surgery, the median V_T_/kgIBW in the conceptual group was lower than that in the non-conceptual group, at 8.2 (8.0–9.2 [7.1–10.3]) vs. 9.2 (9.1–10.1 [7.6–10.1]) mL/kg; (*p* = 0.033).

**Table 1:** Relationship of knowledge regarding lung-protective ventilation strategy with V_T_ and PEEP

	Non-conceptual	Conceptual	*p*-value	OR (95% CI)
No.	8	73		
1st & 2nd year residents	3	13	0.014	
3rd & 4th year residents	0	21		
Fellows	1	21		
Assistant Professors	0	9		
Associate Professors & Professors	4	9		
V_T_ in laparoscopic surgeries (mL/kg IBW)	9.22 (9.07−10.09)	8.20 (7.99−9.22)	0.033	
PEEP in laparoscopic surgeries, n (%)	5/8 (62.5%)	69/72 (95.8%)	0.012	13.80 (2.19−86.88)
V_T_ in non-laparoscopic surgeries (mL/kg IBW)	6.79 (6.10−7.55)	6.92 (6.29−7.61)	0.533	
PEEP in non-laparoscopic surgeries, n (%)	5/8 (62.5%)	62/73 (84.9%)	0.136	3.38 (0.71−16.23)
V_T_ in obese patients (mL/kg IBW)	8.34 (7.1−9.1)	7.58 (7.58−8.49)	0.571	
PEEP in obese patients, n (%)	6/8 (75.0%)	67/71 (94.4%)	0.110	5.58 (0.84−37.02)
Conventional PEEP	4/8 (50%)	65/73 (89.0%)	0.015	8.13 (1.69−38.90)

V_T_, tidal volume; PEEP, positive end-expiratory pressure; OR, odds ratio; IBW, ideal body weight; CI, confidence interval

Anaesthesiologists with knowledge of the concept of lung-protective ventilation (LPV) (conceptual group) applied LPV more often during general anaesthesia than those without knowledge of LPV (non-conceptual group)

The percentage of respondents who applied PEEP in laparoscopic surgery was also higher in the conceptual group than in the non-conceptual group; of the 73 respondents in the conceptual group, 69 responded that they applied PEEP during laparoscopic surgery, while only 5 of the 8 respondents in the non-conceptual group answered that they applied PEEP (69/72 [95.8%] vs. 5/8 [62.5%]; *p* = 0.012; odds ratio [OR] = 13.80 [95% CI, 2.19–86.88]). The conceptual group used PEEP more often than the non-conceptual group in terms of the number of respondents who routinely applied PEEP during anaesthesia (65/73 [89.0%] vs. 4/8 [50.0%]; *p* = 0.015; OR = 8.13 [95% CI, 1.69–38.90]). The level of PEEP (median (interquartile range)) applied in laparoscopic surgery was 5.0 (5.0, 5.0) in both groups. In non-laparoscopic surgery cases, there was no significant difference between the conceptual and non-conceptual groups in terms of median V_T_/kgIBW, at 6.9 (6.3–7.6 [5.9–8.6]) vs. 6.8 (6.1–7.6 [6.0–7.6]) mL/kg (*p* = 0.533), or application of PEEP (62/73 [84.9%] vs. 5/8 [62.5%]; *p* = 0.136; OR = 3.38 [95% CI, 0.71–16.23]; Table 1). The level of PEEP applied in non-laparoscopic surgery was also 5.0 (5.0, 5.0) in both groups. In cases of obese patients, there was no significant difference between the conceptual and non-conceptual groups in terms of median V_T_/kgIBW, at 7.6 (7.6–8.5 [6.3–10.4]) vs. 8.3 (7.1–9.1 [6.4–9.1]) mL/kg (*p* = 0.571), or application of PEEP (67/71 [94.4%] vs. 6/8 [75.0%]; *p* = 0.110; OR = 5.58 [95% CI, 0.84–37.02]. The level of PEEP applied in obese patients in the conceptual group and the non-conceptual group were 5.0 (5.0, 7.0), and 5.0 (5.0, 5.50), respectively (*p* = 0.219).

#### Difference between the correct answer group and the incorrect answer group

Anaesthesiologists who chose the correct answer to a multiple-choice question (correct answer group) applied lower V_T_/kgIBW than those who chose the incorrect answer (incorrect answer group; Table 2). Among cases of laparoscopic surgery, the median V_T_/kgIBW in the correct answer group was lower than that in the incorrect answer group, at 8.4 (7.8–9.2 [6.9–9.8]) vs. 9.6 (8.8–10.2 [8.2–12.1]) mL/kg (*p* <  0.001). In cases of obese patients, the median V_T_/kgIBW in the correct answer group was lower than that in the incorrect answer group, at 7.8 (7.6–8.3 [6.1–9.1]) vs. 8.8 (7.6–9.1 [6.4–12.1]) mL/kg (*p* = 0.014). However, in non-laparoscopic surgery cases, there was no significant difference between the correct answer and incorrect answer groups in terms of median V_T_/kgIBW, at 7.1 (6.3–7.5 [5.7–8.5]) vs. 6.7 (6.0–7.3 [6.0–8.2]) mL/kg (*p* = 0.109). The level of PEEP applied was comparable between the 2 groups for cases of laparoscopic surgery, non-laparoscopic surgery, and obese patients, at 5.0 (5.0, 5.0), 5.0 (5.0, 5.0), and 5.0 (5.0, 7.0), respectively.

**Table 2:** Differences in V_T_ and PEEP settings between the correct and incorrect answer groups

	Non-conceptual	Conceptual	*p*-value	OR (95% CI)
No.	19	63		
1st & 2nd year residents	3	13	0.040	
3rd & 4th year residents	4	17		
Fellows	3	20		
Assistant Professors	1	8		
Associate Professors & Professors	8	5		
V_T_ in laparoscopic surgeries (mL/kg IBW)	9.64 (8.81–10.25)	8.37 (7.79–9.22)	< 0.001	
PEEP in laparoscopic surgeries, n (%)	16/19 (84.2%)	59/62 (95.2%)	0.138	0.27 (0.05–1.47)
V_T_ in non-laparoscopic surgeries (mL/kg IBW)	6.72 (6.04–7.30)	7.06 (6.29–7.55)	0.109	
PEEP in non-laparoscopic surgeries, n (%)	14/19 (73.7%)	54/63 (85.7%)	0.296	2.14 (0.62–7.41)
V_T_ in obese patients (mL/kg IBW)	8.80 (7.58–9.10)	7.81 (7.58–8.34)	0.014	
PEEP in obese patients, n (%)	14/18 (77.8%)	60/62 (96.8%)	0.021	8.57 (1.43–51.56)
Conventional PEEP	13/19 (68.4%)	57/63 (90.5%)	0.027	4.39 (1.22–15.80)

V_T_, tidal volume; PEEP, positive end-expiratory pressure; OR, odds ratio; IBW, ideal body weight, CI, confidence interval

Anaesthesiologists who chose the correct answer to a multiple-choice question (correct answer group) applied lower V_T_/IBW than those who chose an incorrect answer (incorrect answer group)

The correct answer group included more respondents who routinely applied PEEP during anaesthesia than the incorrect answer group (57/63 [90.5%] vs. 13/19 [68.4%]; *p* = 0.027; OR = 4.39 [95% CI, 1.22–15.80]). Of the 62 respondents in the conceptual group, 60 responded that they applied PEEP in obese patients, while 14 of the 18 respondents in the incorrect answer group answered that they applied PEEP (60/62 [96.8%] vs. 14/18 [77.8%]; *p* = 0.021; odds ratio [OR] = 8.57 [95% CI, 1.43–51.56]).

### 2. Retrospective study

Of the 15,982 cases of surgery under general anaesthesia in 2004, 1450 cases involved cardiothoracic surgery, 994 cases involved paediatric surgery, and 12,536 cases had missing or incomplete data; consequently, these cases were all excluded. The 2004 group finally included 1002 cases. Of the 33,538 cases of surgery under general anaesthesia in 2014, 5236 cases involved paediatric surgery, 1245 involved cardiothoracic surgery, and 26,051 cases had missing or insufficient data. Upon excluding these cases, the 2014 group finally comprised 1006 cases.

#### Demographics

After PS-matching of patients between the 2004 and 2014 groups, there were no significant differences in demographic data—including weight, height, age, and sex—between the 2 groups (Table 3). Finally, 818 cases in each group were included for final analysis.

**Table 3:** Comparison of demographic and clinical data between patients who underwent surgery under general anaesthesia in 2004 and 2014

Variable	2004 (*n* = 818)	2014 (n = 818)	*p*-value
Age	50.1 ± 15.1	49.4 ± 16.7	0.320
Sex	372: 446	374: 444	0.921
Weight (kg)	62.4 ± 10.8	62.8 ± 12.3	0.420
Height (cm)	163.3 ± 8.3	163.2 ± 8.3	0.820
ASA class (1 or 2 vs. 3 or 4)	645: 173	649: 169	0.808
BSA (m^2^)	1.68 ± 0.17	1.68 ± 0.19	0.718
Lapa op (%)	83 (10.1%)	132 (16.2%)	< 0.001
Open abd. (%)	224 (27.4%)	70 (8.6%)	
Head & neck (%)	228 (27.9%)	350 (42.8%)	
Orthopaedic (%)	58 (7.1%)	127 (15.5%)	
Urology (%)	44 (5.4%)	46 (5.6%)	
Spine op (%)	69 (8.4%)	17 (2.1%)	
Other op (%)	112 (13.7%)	76 (9.2%)	
Prone position (%)	60 (7.3%)	14 (1.7%)	< 0.001
V_T_ (mL/kgIBW)	9.9 ± 1.4	8.4 ± 1.0	< 0.001
V_T_ (mL/kg of ABW)	9.1 ± 1.3	7.7 ± 1.1	0.001
V_T_ (mL)	559.6 ± 80.9	475.6 ± 59.5	< 0.001
PEEP, N (%)	1 (0.0%)	381 (46.6%)	< 0.001
Lung-protective ventilation, N (%)	0 (0.0%)	280 (34.2%)	< 0.001

BSA, body surface area; V_T_, tidal volume; IBW, ideal body weight; ABW, adjusted body weight; PEEP, positive end-expiratory pressure; Lapa op, laparoscopic surgery; Open abd, open abdominal surgery. Other operations included breast surgery, hernioplasty, hysteroscopy, anal surgeries, plastic surgeries. Values refer to mean (SD) or number (proportion, %)

The patients were categorised according to the type of surgery. The categories of patients in 2004 were as follows: laparoscopic surgery (*n* = 83), open abdominal surgery (*n* = 224), head and neck surgery (*n* = 228), orthopaedic surgery (*n* = 58), urological surgery (*n* = 44), spine surgery (*n* = 69), and other surgeries (*n* = 112). The categories of patients in 2014 were as follows: laparoscopic surgery, (*n* = 132), open abdominal surgery (*n* = 70), head and neck surgery (*n* = 350), orthopaedic surgery, (*n* = 127), urological surgery (*n* = 46), spine surgery (*n* = 17), and other surgeries (*n* = 76).

#### Adoption of LPV

There was a significant increase in the number of patients receiving LPV (V_T_ < 10 mL/kgIBW and PEEP ≥5 cm H_2_O) between 2004 and 2014 (0/818 [0.0%] vs. 280/818 [34.2%]; *p* <  0.001).

#### Tidal volume

**Fig. 2:**
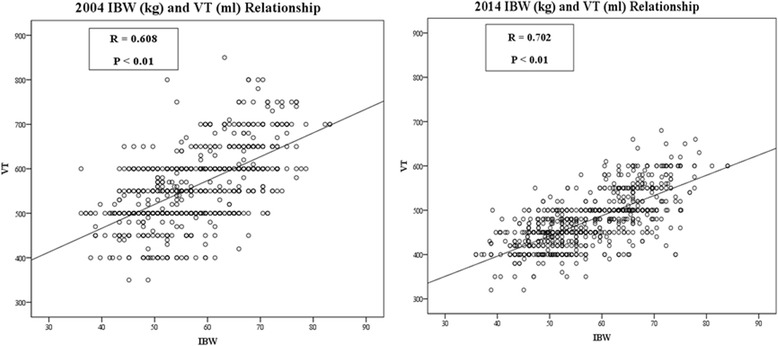
Relationship between ideal body weight (IBW) and tidal volume (V_T_) among patients selected from 2004 and 2014. V_T_ settings in 2004 were higher than those in 2014. R: coefficient of correlation. R^2^: coefficient of determination

The absolute mean V_T_ had decreased over a span of 10 years (2004 vs. 2014: 559.6 ± 80.9 vs. 475.6 ± 59.5 mL; *p* <  0.001). Similarly, the mean V_T_/kgIBW had reduced between 2004 and 2014 (9.9 [1.4] vs. 8.4 [1.0] mL/kg; *p* <  0.001; Table 3). Between 2004 and 2014, there was a significant reduction in the number of patients receiving ventilation with V_T_ > 10 mL/kgIBW (356/818 [43.5%] vs. 48/818 [5.9%]; p <  0.001). In both 2004 and 2014, there was a significant correlation between V_T_ and IBW (*p* <  0.01), with a stronger correlation in 2014 (R^2^: 0.49 vs. 0.37; Fig. 2).

In obese patients (BMI > 30 kg/m^2^) [28], the mean V_T_ had decreased over the 10-year span (2004 vs. 2014: 583.1 ± 99.1 vs. 504.5 ± 74.5 mL; *p* <  0.001); the mean V_T_ / kgIBW also exhibited a similar reduction (10.7 ± 1.5 vs. 8.9 ± 0.9 mL/kg; p <  0.001).

Upon regression analysis, in both 2004 and 2014, height, weight, and male sex were the risk factors that increased the V_T_. The prone position was not associated with V_T_. In contrast, laparoscopic surgery was the factor associated with a decrease in V_T,_ as compared to non-laparoscopic surgery, in 2004; however, this factor did not affect V_T_ in 2014 (Table 4).

**Table 4:** Comparison of factors associated with tidal volume among patients who underwent surgery under general anaesthesia in 2004 and 2014

	Tidal Volume (2004)		Tidal Volume (2014)	
	Beta	p-value	Beta	*p*-value
Type of surgery: Laparoscopic surgery (vs. non-laparoscopic surgery)	−22.09	0.001	−.6.15	0.319
Posture: Prone position (vs. other than prone position)	11.57	0.145	1.75	0.909
Sex: Female (vs. male)	−22.57	0.003	−29.66	0.001
Age	−0.19	0.203	−0.31	0.052
Weight	2.86	< 0.001	1.98	< 0.001
Ideal body weight	2.36	< 0.001	1.80	0.001
R^2^	0.477		0.621	

In 2004, V_T_ was associated with laparoscopic surgery as well as sex, height, and weight; in contrast, in 2014, only sex, height, and weight were associated with V_T_. Prone position was not associated with V_T_

Beta: regression coefficient

R^2^: coefficient of determination

#### PEEP

The usage of PEEP had significantly increased between 2004 and 2014 (1/818 [0.0%] vs. 381/818 [46.6%]; p <  0.001; Table 3); this trend was also observed among obese patients (0/32 [0%] vs. 21/38 [55.3%]; *p* < 0.001). The median PEEP values (interquartile range) of all patients and of obese patients in 2014 were 0.0 (0.0−5.0) and 5.0 (0.0−5.0), respectively. There was no significant difference in the usage of PEEP between patients who underwent laparoscopic and open abdominal surgery in 2014 (57/124 [46.0%] vs. 36/66 [54.5%]; *p* = 0.260). The median PEEP values (interquartile range) in laparoscopic surgery and open abdominal surgery in 2014 were 5.0 (0.0−5.0) and 5.0 (3.0−5.0), respectively.

## Discussion

The main finding of this study was that anaesthesiologists with cognizance of LPV applied LPV more often during general anaesthesia than those without cognizance of LPV. These results suggest that the knowledge of LPV strategy is directly related to its implementation, and can explain the pattern of increased LPV use in general anaesthesia. Therefore, this study provide further supportive evidence for the effect of education and feedback that decreased the average intraoperative tidal volume and improved the rate of LPV use [21].

We conducted a questionnaire survey on the assumption that knowledge of LPV among anaesthesiologists would affect implementation of LPV in the operating room. The majority of anaesthesiologists surveyed in 2016 responded that they knew about LPV. Most of the respondents who replied that they did not know about LPV were first- and second-year residents and senior anaesthesiologists. The conceptual and non-conceptual groups exhibited significant differences in V_T_ and PEEP settings in laparoscopic surgery. The 2 groups also differed in terms of routine application of PEEP; however, there were no statistically significant differences in V_T_ and PEEP settings during non-laparoscopic surgery and in cases of obese patients.

Because V_T_ in LPV is based on IBW [22–24], and IBW is determined by sex and height, respondents who chose ‘height’ to question 5 (a multiple-choice question) could be considered to understand the concept of IBW. The percentage of correct answers for this question was high (63/82 [76.85%]), and senior anaesthesiologists had the lowest percentage of correct answers for this multiple-choice question (5/13 [38.5%]). The correct answer group and incorrect answer group (in terms of this question) also exhibited significant differences in V_T_/kgIBW settings in laparoscopic surgery and in cases of obese patients. However, there were no statistically significant differences in V_T_/kgIBW settings during non-laparoscopic surgery.

Overweight and obese patients are more often exposed to greater V_T_ than patients with normal body weight. Therefore, a greater awareness for appropriate selection of V_T_ on the basis of IBW is highly recommended in such patients [13]. The ‘obese patient’ in our questionnaire was an extremely obese patient with a BMI of 41.52, and the correct answer group applied a relatively low V_T_, even in this patient. Several studies have reported that LPV might also reduce pulmonary complications in laparoscopic surgery [10, 14, 29]. It has been shown that LPV can reduce barotrauma and lung inflammation and improve postoperative oxygenation even for patients operated on in the prone position [15, 30]. Both the conceptual group and the correct answer group applied lower V_T_ than the non-conceptual group and the incorrect answer group in cases of laparoscopic surgery. In contrast, the patient undergoing non-laparoscopic surgery in our questionnaire was a thin patient with a BMI of 18.12, and thus there was no significant difference in V_T_/kgIBW according to cognizance of LPV strategy. These results suggest that the knowledge of LPV strategy is directly related to the application of LPV in general anaesthesia. Lack of education and knowledge might be obstacles to the application of LPV in practice.

Anaesthesiology residency training at our institution includes an intensive care unit (ICU) course. In the ICU, LPV is the standard treatment for ARDS; this strategy is also widely applied in patients with conditions other than ARDS. Residents are repeatedly instructed to set a V_T_ of 6–8 mL/kgIBW during mechanical ventilation in critically ill patients. Additionally, each ventilator carries a chart with pre-calculated values of V_T_/kgIBW, which has been reported to be very useful in preventing high-V_T_ ventilation [31]. It is presumed that this training process would have affected the ventilation practice in the operating room. However, most of the attending anaesthesiologists in our institution are not attending physicians at the ICU, but are dedicated to the operating room. Consequently, they might not have had the opportunity to gain knowledge regarding LPV.

According to our retrospective study, even in 2014, the LPV strategy was not fully implemented in the operating room at our institution. However, application of LPV has definitely increased [20]. There is accumulating evidence regarding the effectiveness of LPV during general anaesthesia [9, 32]. Recent meta-analyses of randomised controlled trials demonstrated that, relative to surgery without LPV (high V_T_ [> 10 mL/kg] and no PEEP), intraoperative LPV strategies involving low V_T_ (6–8 mL/kgIBW), high PEEP (> 5 cmH_2_O), and intermittent recruitment manoeuvres were associated with a statistically significant reduction in the incidence of postoperative atelectasis, lung infection, and acute lung injury [1, 26, 33]. Because such knowledge is becoming universal, the adoption of an LPV strategy has increased.

There is typically a delay in dissemination of knowledge from the time of discovery of new evidence to its implementation in clinical practice [34]. The current level of education and knowledge could be a contributing factor to this gap between the theoretical best practice and its practical application. There has been a lack of rapid and widespread adoption of the LPV strategy in ARDS treatment, where previous studies have demonstrated variations in practice with experience, knowledge, and position of the clinician [35–37].

In fact, previous studies have reported that anaesthetic induction skills—including tracheal intubation and arterial and central line catheterisation—could be improved by gaining experience and education through workshops [38–40]. Additionally, in LPV strategies, a knowledge deficit regarding the use of low-V_T_ for ARDS is common and varies according to the type and experience of the caregiver. A survey-based study on low-V_T_ ventilation in patients with ARDS reported lower perception of barriers and higher knowledge-test scores among fellows and attending physicians than among interns and residents [37]. Previous studies have also demonstrated that usage of a low-V_T_ strategy increases after feedback and education involving presentation of actual ventilation settings and discussion on potential reasons for not using low-V_T_ [36, 41, 42]. In a recent study, as in the ICU, education and feedback was found to be necessary for adoption of LPV in general anaesthesia [21].

In our retrospective study, the percentage of cases involving intraoperative LPV (V_T_ < 10 mL/kgIBW and PEEP ≥5 cmH_2_O) had significantly increased over a span of 10 years. These results correspond with those of earlier studies. In a 5-year retrospective study, Hess et al. reported a reduction in the percentage of patients receiving ventilation with V_T_ > 10 mL/kgIBW and without PEEP during general anaesthesia [18].

In the present study, the mean V_T_/kgIBW among obese patients (BMI > 30 kg/m^2^) had significantly reduced between 2004 and 2014, while the usage of PEEP in this subgroup had significantly increased. The results of regression analysis revealed a significant difference in the factors affecting V_T_ settings between the 2 study periods. In 2004, V_T_ was associated with laparoscopic surgery as well as sex, height, and weight; in contrast, in 2014, only sex, height, and weight were associated with V_T_. Prone position was not associated with V_T_.

In 2004, anaesthesiologists tended to set lower V_T_ during laparoscopic surgery than during other open surgeries (530.5 [69.3] vs. 553.6 [69.6] mL; *p* = 0.010). A possible explanation for this trend is that the V_T_ was inevitably set low for laparoscopic surgery, where the peak inspiratory pressure increases markedly [43, 44]. In contrast, in 2014, there was no difference in V_T_ between laparoscopic and open abdominal surgery (482.3 [62.8] vs. 486.4 [64.0] mL; *p* = 0.660). The knowledge of LPV has been accepted by anaesthesiologists, and the LPV strategy has been applied more frequently in open surgery. The variation in ventilator settings during open surgery seems to have reduced with the increase in number of and familiarity with laparoscopic surgeries over a span of 10 years [45]. These trends may be interpreted as reflecting an improved cognizance of LPV in general anaesthesia.

The present study has some limitations. First, the questionnaires were given to predominantly junior anaesthesiologists, of which most would have trained in the era of LPV. The answers given to the questions and the actual practice may differ [37], in that the decision on the patient management would depend on senior anaesthesiologists. Secondly, the definition of LPV (V_T_ < 10 mL/kgIBW and PEEP ≥5 cmH_2_O) in our study—although based on previous studies [20, 25, 26] in patients without acute lung injury undergoing general anaesthesia—is arbitrary and differs from the standard ARDS treatment guidelines. Thirdly, this retrospective study involves many uncontrolled co-factors—including fluid intake, operation time, blood products, and type of surgery and intravenous fluid—which cannot be controlled in this type of study. Therefore, we only used the initial ventilator settings for analysis. Finally, this study only involved a single centre in South Korea. Consequently, respondents in this questionnaire study probably do not represent the larger population of anaesthesiologists. In this retrospective study, it is not possible to determine whether our results are applicable to another institution in South Korea. Nevertheless, this questionnaire study is meaningful in that we achieved complete enumeration of the majority of anaesthesiologists who have been in charge of anaesthesia for more than a decade participated in the survey, and the responses of anaesthesiologists on all levels were used in the analysis. It is also important to note that there have been few studies on the relationship of cognizance of LPV and adoption of LPV strategy in general anaesthesia. The present results provide clues for understanding the changes in anaesthetic methods, including LPV, during general anaesthesia in South Korea.

## Conclusions

In summary, in a questionnaire survey, we found that anaesthesiologists with cognizance of LPV applied LPV more often during general anaesthesia than those without cognizance of LPV. This finding explains the results of our retrospective study, which demonstrated that adoption of LPV during general anaesthesia increased significantly over a period of 10 years. Further studies assessing the impact of intraoperative LPV on clinical outcome are required, and more research to determine the efficacy of intraoperative LPV education is needed.

## Additional file

Additional file 1: Questionnaire for the setting up of intraoperative respiratory parameters. (DOCX 12 kb)

## Abbreviations

IBW: Ideal body weight
ICU: Intensive care unit
IQR: Interquartile range
LPV: Lung-protective ventilation
PEEP: Positive end expiratory pressure
SD: Standard deviation
V_T_: Tidal volume
