# Supplementary material for: Application of intraoperative lung-protective ventilation varies in accordance with the knowledge of anaesthesiologists: a single-Centre questionnaire study and a retrospective observational study
Source: BMC Anesthesiol. 2018 Apr 2;18:33. doi: 10.1186/s12871-018-0495-7 (PMC5879938; doi:10.1186/s12871-018-0495-7)
Supplement: Supplementary file 1 — Questionnaire for the setting up of intraoperative respiratory parameters. (DOCX 12 kb) [file 12871_2018_495_MOESM1_ESM.docx]

**Questionnaire for the setting up of intraoperative respiratory parameters**

1. Set the initial ventilator settings for patients without respiratory disease.

A 50-year-old woman scheduled for laparoscopic hysterectomy; height: 156 cm; weight: 85 kg.

Tidal volume: mL.

Positive end-expiratory pressure: Yes or No; in case ‘Yes’, cmH_2_O.

2. Set the initial ventilator settings for patients without respiratory disease.

A 45-year-old man scheduled to undergo surgery for clavicle fracture; height: 185 cm; weight: 62 kg.

Tidal volume: mL.

Positive end-expiratory pressure: Yes or No; in case ‘Yes’, cmH2O.

3. Set the initial ventilator settings for patients without respiratory disease.

A 66-year-old man scheduled for bariatric surgery; height: 170 cm; weight: 120 kg

Tidal volume: mL.

Positive end-expiratory pressure: Yes or No; in case ‘Yes’, cmH2O.

4. During the initial setting for ventilation, do you routinely set the end-tidal positive pressure?

5. In the initial setting for mechanical ventilation, what variable do you consider most important?

① sex

② height

③ total body weight

④ whether surgery is laparoscopic or not

⑤ patient position

6. Do you know about the lung-protective ventilation strategy? Yes or No
